# The Long Haul to Surgery: Long COVID Has Minimal Burden on Surgical Departments

**DOI:** 10.3390/ijerph21091205

**Published:** 2024-09-12

**Authors:** Nicole Hamilton Goldhaber, Karthik Ramesh, Lucy E. Horton, Christopher A. Longhurst, Estella Huang, Santiago Horgan, Garth R. Jacobsen, Bryan J. Sandler, Ryan C. Broderick

**Affiliations:** 1 Department of Surgery, School of Medicine, University of California San Diego, La Jolla, San Diego, CA 92037, USA; 2 School of Medicine, University of California San Diego, La Jolla, San Diego, CA 92093, USA; 3 Division of Infectious Diseases and Global Public Health, Department of Medicine, School of Medicine, University of California San Diego, La Jolla, San Diego, CA 92037, USA; 4 Division of Biomedical Informatics, Department of Medicine, School of Medicine, University of California San Diego, La Jolla, San Diego, CA 92037, USA; 5 Department of Pediatrics, School of Medicine, University of California San Diego, La Jolla, San Diego, CA 92037, USA

**Keywords:** long COVID, post-acute sequelae of SARS-CoV-2, surgical burden, pandemic, COVID-19

## Abstract

Many patients infected with the SARS-CoV-2 virus (COVID-19) continue to experience symptoms for weeks to years as sequelae of the initial infection, referred to as “Long COVID”. Although many studies have described the incidence and symptomatology of Long COVID, there are little data reporting the potential burden of Long COVID on surgical departments. A previously constructed database of survey respondents who tested positive for COVID-19 was queried, identifying patients reporting experiencing symptoms consistent with Long COVID. Additional chart review determined whether respondents had a surgical or non-routine invasive procedure on or following the date of survey completion. Outcomes from surgeries on patients reporting Long COVID symptoms were compared to those from asymptomatic patients. A total of 17.4% of respondents had surgery or a non-routine invasive procedure in the study period. A total of 48.8% of these patients reported experiencing symptoms consistent with Long COVID. No statistically significant differences in surgical outcomes were found between groups. The results of this analysis demonstrate that Long COVID does not appear to have created a significant burden of surgical disease processes on the healthcare system despite the wide range of chronic symptoms and increased healthcare utilization by this population. This knowledge can help guide surgical operational resource allocation as a result of the pandemic and its longer-term sequelae.

## 1. Introduction

Long COVID, also known as Post-Acute Sequelae of SARS-CoV-2 infection (PASC), has emerged as a significant medical concern in the aftermath of the COVID-19 pandemic. Many patients infected with the virus continue to experience symptoms or begin to experience new symptoms for weeks to years as sequelae of the initial infection; thus, this is referred to as “Long COVID” [1]. According to the Centers for Disease Control and Prevention (CDC), as of June 2022, nearly 1 in 5 adults in the United States (US) who have had COVID-19 still have Long COVID, and 1 in 13 adults in the US overall have Long COVID symptoms [2]. Given these estimates of COVID-19 survivors experiencing long-term health consequences, Long COVID poses a considerable burden on healthcare systems worldwide.

There are a broad range of symptoms associated with Long COVID as there are with initial COVID-19 infection [3,4,5,6,7], ranging from shortness of breath and loss of taste or smell to generalized weakness, fatigue, and neurologic symptoms such as forgetfulness and difficulty concentrating that are often referred to as “brain fog”. To help categorize and assess resource support for patients suffering from this condition, the authors of this work previously described presentation of multiple clusters of patient symptoms or sequelae resulting from Long COVID, including gastrointestinal (GI), musculoskeletal (MSK), neurocognitive (NCG), airway (AW), and cardiopulmonary (CP) symptoms [8].

Surgical departments are not exempt from the challenges presented by Long COVID. Surgical volume throughout the COVID-19 pandemic consistently decreased in early and middle 2020, with some rebounding volume thereafter [9,10,11,12,13]. However, these studies do not specifically address possible relationships between Long COVID and surgical utilization or how they might contribute to these trends. As elective surgeries resume and the demand for surgical interventions continues to grow, understanding the implications of Long COVID on surgical burden and outcomes is of paramount importance.

As our society continues to face this complex and evolving clinical challenge, there is still an open question regarding the impact of Long COVID itself on surgical procedure rates and outcomes. A comprehensive analysis of the burden of Long COVID on the Surgical Department at a quaternary academic medical center, including utilization of surgical services by Long COVID patients, and perioperative outcomes is included in this work. We hypothesize that there will not be a significant difference in the utilization of resources by Long COVID patients compared to patients who did not report Long COVID symptoms.

## 2. Materials and Methods

A database was previously constructed from the results of a population-based electronic survey of patients who tested positive for COVID-19 at the study institution between 1 March 2020 and 1 July 2021 as described by Goldhaber et al. [1]. The survey was sent to patients at least 90 days from their initial infection date, and it was utilized to identify patients who reported experiencing symptoms consistent with Long COVID and to characterize their symptom profile [8]. The current project is a secondary use extension of an operational project previously approved for Institutional Review Board exemption by the University of California San Diego Health Human Research Protection Program.

Additional chart reviews were then conducted to determine if survey respondents had undergone a surgical or non-routine invasive procedure on or following the date of survey completion, as well as for information regarding the initial perioperative outcomes of those surgical procedures. The following variables were queried: case type, surgical service line, case urgency (emergent, urgent/time-sensitive, or elective), inpatient status after surgery, postoperative destination (intensive care unit [ICU] or non-ICU), hospital length of stay after surgery, and 30-day complication rate.

The burden of Long COVID on the study institution’s surgery department was determined through comparison of the number of surgeries performed on patients who reported having Long COVID symptoms to those on patients who did not report having symptoms. Statistical analyses were conducted using Python and the Scipy package including two-tailed Student’s *t*-tests and Fisher’s exact tests. A *p*-value of <0.05 was considered statistically significant.

## 3. Results

Of 9619 (10.4%) patients, 999 responded to the original survey, 421 (46.3%) of whom reported experiencing symptoms consistent with Long COVID. The most commonly reported symptoms included weakness/tiredness, sleep disturbances, and difficulty thinking/concentrating (“brain fog”) [1]. The participants’ sociodemographic and clinical characteristics were previously reported [8] and can be found in Table S1.

Of all the survey respondents, 174 (17.4%) were found to have undergone surgery or a non-routine invasive procedure on or following the date of survey completion (at least 90 days following a positive COVID-19 test). Of these patients, 85 (48.8%) were those who reported experiencing symptoms consistent with Long COVID. Differences in outcome variables, including case urgency (emergent, urgent/time-sensitive, or elective), inpatient status after surgery, postoperative destination (ICU or non-ICU), hospital length of stay after surgery, and 30-day complication rate, are reported in Table 1. Overall, 76 (43.7%) patients underwent emergent/urgent/time-sensitive procedures (43.5% of Long COVID patients who underwent procedures versus 43.8% of asymptomatic patients who underwent procedures), as opposed to elective procedures. This difference is not statistically significant (*p* = 0.97).

**Figure 1:**
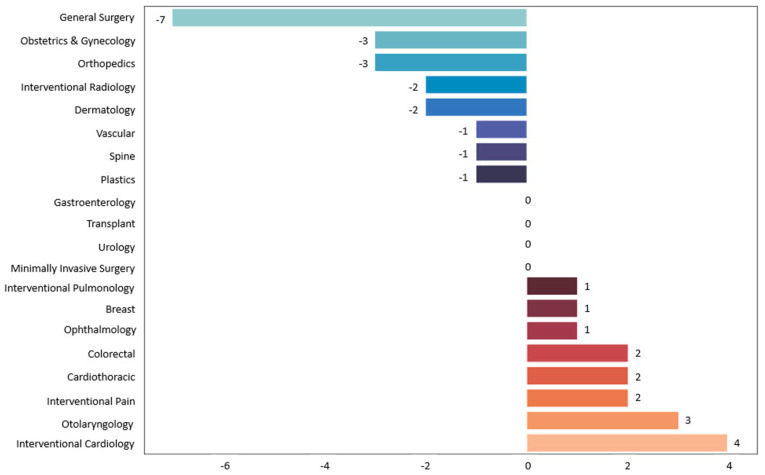
Difference between number of operations by specialty between group of patients with Long COVID and group of patients without Long COVID. Negative numbers indicate greater number of procedures in group without Long COVID symptoms, and positive numbers indicate greater number of procedures in group with Long COVID symptoms.

There were no statistically significant differences between groups who underwent surgeries in the study period, pertinently in the percentage of emergent or inpatient cases, those with postoperative ICU stays, in hospital length of stay, or in the rate of complications 30 days postoperatively. An additional descriptive analysis was conducted of the count of cases by the surgical department, shown in Figure 1. There were also no statistically significant differences in these comparisons; though notably, in the study cohort, Cardiothoracic Surgery, Interventional Cardiology, and Interventional Pulmonology cases were only logged in patients who reported symptoms of Long COVID, and Interventional Pain and ENT had greater than or equal to two more cases in Long COVID patients compared to the other group, while Orthopedics, Dermatology, and OBGYN had greater than or equal to two or more cases in the group that did not report Long COVID symptoms. The remainder of the services were within one or fewer cases between groups.

## 4. Discussion

In this study, we aimed to determine the burden of Long COVID on surgical departments by comparing the incidence of surgical cases among patients with and without self-reported Long COVID symptoms. Despite the growing awareness of the long-term health impacts of Long COVID and the increased risk for developing other chronic conditions, our findings revealed no statistically significant differences between the surgical outcomes for patients with and without Long COVID symptoms.

Healthcare systems have been impacted significantly by the COVID-19 pandemic and substantial numbers of Long COVID patients are seeking medical and surgical care [14]. With prevalence as high as that being reported by the CDC (20% of COVID-19 survivors) [2], as high as other reports in the literature (5–50%) [15,16,17], or as high as found in COVID-19 survivors who responded to our institution’s survey (46.3%) [1,8], it is clear that Long COVID is causing a significant burden on our society and healthcare system as a whole.

Others have sought to understand the impact of this condition on patients’ utilization of medical services. The findings suggest that acute COVID illness is associated with increased healthcare utilization, including the utilization of surgical services [11,13]. However, these findings have not, as of yet, pursued a case–control methodology nor included chart reviews or patient interviews to determine the persistence of Long COVID-like symptoms and thus cannot clearly identify the causality of persistence of symptoms with surgical or other healthcare utilization [14].

One known impact of the COVID-19 pandemic on surgical departments is delayed presentation of many conditions or diseases. As many as 40% of adults have reportedly delayed medical care due to COVID-19-related concerns, including urgent or emergent care in greater than 10% of these cases [18]. This includes surgical cases, with an estimated decrease in volume of around 50% after March 2020 [19]. While the surgeries that were canceled were primarily elective, thus protecting patients from exposure to COVID-19, this allowed time for disease progression and potentially worse outcomes [20]. An example of this is the delayed presentation of appendicitis, which increased the incidence of suppurative and gangrenous appendicitis by 17% and 7%, respectively, in one study [21]. This potentially leads to increased complications or hospital stay and an increased burden on the healthcare system. However, these phenomena have not been directly connected with Long COVID.

There are several potential limitations in the generalizability of the results of the current study. First, the lack of a universally accepted definition of and diagnostic criteria for Long COVID may have impacted our ability to accurately identify affected individuals. Moreover, we are only able to identify surgeries or procedures that occur at the study institution or for which records are accessible through the study institution’s electronic health record system (EHR). Additionally, the self-reported nature of our survey might have introduced recall bias and over- or under-reporting of symptoms, potentially affecting this study’s findings. The relatively small sample size of specifically surgical patients (n = 174) represents another limitation of this study, as a larger cohort would have increased the statistical power to potentially detect subtle differences in surgical outcomes between groups. Matching between groups based on sociodemographic or other potentially confounding factors (including but not limited to age, sex, comorbidities, and socioeconomic status, which could affect both the presence of Long COVID symptoms and surgical outcomes) was not performed in this analysis in order to preserve the sample size and maintain statistical power. Further investigation with larger cohorts is warranted.

The absence of statistically significant differences between the groups may also reflect the resilience and adaptability of surgical departments in addressing the challenges posed by Long COVID. The experience gained during the COVID-19 pandemic has led to the development and implementation of various strategies to optimize patient care and surgical outcomes [13]. For example, an institution reported implementing a phased approach to reductions in the number of operating rooms in line with the American College of Surgery’s guidelines for triage of nonemergent surgery [22]. As a result, surgical departments may have become more adept at managing patients with complex medical conditions, including Long COVID.

Additionally, the preoperative optimization strategies employed by surgical departments might have mitigated the potential impact of Long COVID on surgical outcomes. In recent years, there has been a concerted effort to improve preoperative assessment of and optimization for patients undergoing elective surgical or invasive procedures, including multi-disciplinary input and targeted interventions for high-risk patients [23,24,25]. Such measures could have helped address COVID-19-related risks, as well as Long COVID-related risks, potentially resulting in comparable outcomes in patients with and without Long COVID symptoms.

It is also worth considering that the surgical procedures included in this study might not have been equally affected by Long COVID. Some surgeries may inherently carry a lower risk of Long COVID-related complications, while others might be more susceptible. Interestingly, the descriptive analysis of department makeup of procedures in both groups of patients suggests notably increased utilization of Cardiothoracic and Interventional Cardiology services among patients with Long COVID symptoms, although this was not statistically significant in this case, likely due to the underpowered analysis due to the small sample size. However, further statistical investigation of the potential significance of these differences is hampered here due to the small population sizes across all the departments. The authors of this study were also limited by inconsistent records in their institution’s EHR of surgeries that may been performed at other institutions.

The results of this analysis demonstrate that Long COVID does not appear to have created a significant burden of surgical disease processes on and surgical resource utilization within the healthcare system. However, given the limitations of this study and the ongoing evolution of our understanding of Long COVID, further research is warranted. This knowledge can help guide operational resource allocation as a result of the pandemic and its longer-term sequelae, ultimately contributing to the ongoing efforts to optimize patient care and improve surgical outcomes in the face of this complex and evolving clinical and public health challenge.

## 5. Conclusions

Long COVID should be treated and resourced for in a manner similar to other diseases or chronic comorbidities, as there were no increased utilization rates of surgical resources nor adverse outcomes identified.

## Figures and Tables

**Table 1:** Comparisons between surgical procedure characteristics and outcomes in patients with Long COVID symptoms and patients without Long COVID symptoms.

	Total Number Surgical Patients	n (%) Emergent/Urgent Cases	N (%) Inpatient Cases	N (%) ICU * Postop. Destination	Length of Stay (Mean)	N (%) Complications w/in 30 Days Postop
Long COVID ** symptoms reported	85	37 (43.5%)	21 (24.7%)	4 (4.7%)	5.05 days	4 (4.7%)
No Long COVID ** symptoms reported	89	39 (43.8%)	26 (29.2%)	1 (1.1%)	2.77 days	9 (10.1%)
*p-Value*	--	*0.97*	*0.61*	*0.20*	*0.24*	*0.25*

* ICU: intensive care unit; ** COVID: SARS-CoV-2 virus.

## Data Availability

The data presented in this study are available on request from the corresponding author.

## Supplementary Materials

The following supporting information can be downloaded at https://www.mdpi.com/article/10.3390/ijerph21091205/s1, Table S1: Participants’ sociodemographic and clinical characteristics.

## References

[1] Goldhaber N.H., Ogan W.S., Greaves A., Tai-Seale M., Sitapati A., Longhurst C.A., Horton L.E. (2022). Population-based evaluation of post-acute COVID-19 chronic sequelae in patients who tested positive for SARS-CoV-2. Open Forum. Infect. Dis..

[2] Centers for Disease Control and Prevention (CDC) (2022). Nearly One in Five American Adults Who Have Had COVID-19 Still Have “Long COVID”.

[3] Crook H., Raza S., Nowell J., Young M., Edison P. (2021). Long COVID—Mechanisms, Risk Factors, and Management. BMJ.

[4] Lopez-Leon S., Wegman-Ostrosky T., Perelman C., Sepulveda R., Rebolledo P.A., Cuapio A., Villapol S. (2021). More than 50 long-term effects of COVID-19: A systematic review and meta-analysis. Sci. Rep..

[5] Davis H.E., Assaf G.S., McCorkell L., Wei H., Low R.J., Re’em Y., Redfield S., Austin J.P., Akrami A. (2021). Characterizing long COVID in an international cohort: 7 months of symptoms and their impact. EClinicalMedicine.

[6] Nalbandian A., Sehgal K., Gupta A., Madhavan M.V., McGroder C., Stevens J.S., Cook J.R., Nordvig A.S., Shalev D., Sehrawat T.S. (2021). Post-acute COVID-19 syndrome. Nat. Med..

[7] Soriano J.B., Murthy S., Marshall J.C., Relan P., Diaz J.V. (2022). A clinical case definition of post-COVID-19 condition by a Delphi consensus. Lancet Infect. Dis..

[8] Goldhaber N.H., Kohn J.N., Ogan W.S., Sitapati A., Longhurst C.A., Wang A., Lee S., Hong S., Horton L.E. (2022). Deep Dive into the Long Haul: Analysis of Symptom Clusters and Risk Factors for Post-Acute Sequelae of COVID-19 to inform clinical care. Int. J. Environ. Res. Public Health.

[9] Moss W.D., Pires G.R., Samlowski E., Webb J., DeAngelo M.M., Eddington D., Brintz B.J., Agarwal J., Kwok A.C. (2022). Characterizing the volume of surgery and post-operative complications during the COVID-19 pandemic. Arch. Surg..

[10] Angelo J., Soto M., Dai D., Spector D., Orav E.J., Tavakkoli A., Tsai T.C. (2023). Impact of the COVID-19 Pandemic on Inpatient and Outpatient Utilization of Bariatric Surgery. Surg. Endosc..

[11] Soreide K., Hallet J., Matthews J.B., Schnitzbauer A.A., Line P.D., Lai P.B.S., Otero J., Callegaro D., Warner S.G., Baxter N.N. (2020). Immediate and long-term impact of the COVID-19 pandemic on delivery of surgical services. Br. J. Surg..

[12] O’Reilly-Shah V.N., Van Cleve W., Long D.R., Moll V., Evans F.M., Sunshine J.E., Kassebaum N.J., Harrison E.M., Jabaley C.S. (2020). Impact of COVID-19 response on global surgical volumes: An ongoing observational study. Bull. World Health Organ..

[13] Ghoshal S., Rigney G., Cheng D., Brumit R., Gee M.S., Hodin R.A., Lillemoe K.D., Levine W.C., Succi M.D. (2022). Institutional Surgical Response and Associated Volume Trends Throughout the COVID-19 Pandemic and Postvaccination Recovery Period. JAMA Open.

[14] Koumpias A.M., Schwartzman D., Fleming O. (2022). Long-haul COVID: Healthcare utilization and medical expenditures 6 months post-diagnosis. BMC Health Serv. Res..

[15] Ayoubkhani D., Pawelek P., Gaughan C. (2021). Technical Article: Updated Estimates of the Prevalence of Post-Acute Symptoms among People with Coronavirus (COVID-19) in the UK: 26 April 2020 to 1 August 2021.

[16] Carfì A., Bernabei R., Landi F. (2020). Persistent symptoms in patients after acute COVID1-19. JAMA.

[17] Thompson E.J., Williams D.M., Walker A.J., Mitchell R.E., Niedzwiedz C.L., Yang T.C., Huggins C.F., Kwong A.S.F., Silverwood R.J., Di Gessa G. (2022). Long COVID burden and risk factors in 10 UK longitudinal studies and electronic health records. Nat. Commun..

[18] Czeisler M.E., Marynak K., Clarke K.E.N., Salah Z., Shakya I., Thierry J.M., Ali N., McMillan H., Wiley J.F., Weaver M.D. (2020). Delay or Avoidance of Medical Care Because of COVID-19-Related Concerns--United States, June 2020. MMWR Morb. Mortal. Wkly. Rep..

[19] Mattingly A.S., Rose L., Eddington H.S., Trickey A.W., Cullen M.R., Morris A.M., Wren S.M. (2021). Trends in US Surgical Procedures and Health Care System Response to Policies Curtailing Elective Surgical Operations During the COVID-19 Pandemic. JAMA Open.

[20] Fu S.J., George E.L., Maggio P.M., Hawn M., Nazerali R. (2020). The Consequences of Delaying Elective Surgery: Surgical Perspective. Ann. Surg..

[21] Bickel A., Ganam S., Shakra I.A., Farkash I., Francis R., Karra N., Merei F., Cohen I., Kakiashvili E. (2022). Delayed diagnosis and subsequently increased severity of acute appendicitis (compatible with clinical-pathologic grounds) during the COVID-19 pandemic: An observational case-control study. BMC Gastroenterol..

[22] Brumit R., Daily B., Levine W.C. (2020). Surgical case deferment during a global pandemic. J. Med. Syst..

[23] Pfeifer K.J., Selzer A., Whinney C.M., Rogers B., Naik A.S., Regan D., Mendez C.E., Urman R.D., Mauck K. (2021). Preoperative Management of Gastrointestinal and Pulmonary Medications: Society for Perioperative Assessment and Quality Improvement (SPAQI). Mayo Clin. Proc..

[24] Sharma S., Arora L. (2020). Anesthesia for the morbidly obese patient. Anesthesiol. Clin..

[25] Cui H.W., Turney B.W., Griffiths J. (2017). The Preoperative Assessment and Optimization of Patients Undergoing Major Urological Surgery. Curr. Urol. Rep..

