# Supplementary material for: The Long Haul to Surgery: Long COVID Has Minimal Burden on Surgical Departments
Source: Int J Environ Res Public Health. 2024 Sep 12;21(9):1205. doi: 10.3390/ijerph21091205 (PMC11431659; doi:10.3390/ijerph21091205)
Supplement: Supplementary file 1 [file ijerph-21-01205-s001.zip › ijerph-3156912-supplementary.pdf]

Supplementary Material

Table S1. Participant sociodemographic and clinical characteristics. [8].

	No Long COVID Symptoms Reported (n = 488)	Long COVID Symptoms Reported (n = 421)	Test Statistics (99% CI)
Age (years)	51.4 (16.3)	52.2 (15.0)	*c* 0.05 (−0.12–0.22)
Race (%White/Black/AAPI/Other/NR)	56.8 / 4.7 / 9.2 / 19.7 / 9.6	50.8 / 4.0 / 10.7 / 24.7 / 9.7	$X^2_4 = 4.80$, $p > 0.01$
Sex (% female)	56.4%	63.4%	**OR = 1.34 (1.02–1.77)**
HPI (percentile score)	58% (24.8)	56.6 (27.5)	*d* = −0.06 (−0.23–0.12)
Acute COVID-19 hospitalization	6.8%	24.7%	**OR = 4.53 (2.59–8.22)**
Pre-COVID Self-Rated Health	4.27 (0.74)	4.04 (0.80)	***d* = −0.29 (−0.46–−0.12)**
Post-COVID Self-Rated Health	4.24 (0.73)	3.25 (0.91)	***d* = −1.21 (−1.39–−1.02)**
Monoclonal antibody treatment	5.9%	11.5%	**OR = 2.05 (1.06–4.07)**
Days since COVID+ result	315 (3.4)	348 (4.5)	***d* = 0.41 (0.23, 0.58)**

Means and standard deviation shown for continuous variables. Test statistics for continuous variables based on two-sample independent *t*-tests, converted to Cohen's *d*, and for categorical variables based on Chi-squared or Fisher's Exact tests, with 99% confidence intervals (CI) shown. Bold indicates that 99% CI does not cross 0 for continuous variables or 1 for categorical variables. Self-rated health based on five-point scale (1 = very poor, 2 = poor, 3 = fair, 4 = good, 5 = excellent). Higher Healthy Places Index (HPI) denotes higher resourced community. AAPI: Asian-American or Pacific Islander. NR: not reported.
